# The Optimal Dietary Sodium or Chloride Level of Broilers Fed a Corn–Soybean Meal Diet from 22 to 42 Days of Age

**DOI:** 10.3390/ani14223173

**Published:** 2024-11-06

**Authors:** Xiaoyan Cui, Feiyu Gao, Weiyun Zhang, Wei Wu, Liyang Zhang, Yun Hu, Shengchen Wang, Tingting Li, Xugang Luo

**Affiliations:** 1 Poultry Mineral Nutrition Laboratory, College of Animal Science and Technology, Yangzhou University, Yangzhou 225009, China; xycui@yzu.edu.cn (X.C.); g1234567_7@163.com (F.G.); 15516412374@163.com (W.Z.); 15170813454@163.com (W.W.); huyun@yzu.edu.cn (Y.H.); shengchenwang@yzu.edu.cn (S.W.); 2 Mineral Nutrition Research Division, State Key Laboratory of Animal Nutrition, Institute of Animal Science, Chinese Academy of Agricultural Sciences, Beijing 100193, China; zhangliyang@caas.cn

**Keywords:** sodium and chloride level, broiler, growth performance, blood gas indicator, serum indicator

## Abstract

The objective of this study was to assess the ideal dietary Na or Cl level for chickens from 22 to 42 d of age, utilizing a corn–soybean meal diet that maintains a 1:1 ratio of Na to Cl. At 22 d of age, a total of 288 male broilers of Arbor Acres were selected and randomly divided into one of six treatments. Each treatment contained eight replicates, with six chicks in each replicate. The control chicks were given a basic diet including 0.02% Na and 0.07% Cl without added Na or Cl, while the birds in other treatments were given diets containing different Na or Cl levels (0.13%, 0.19%, 0.25%, 0.31%, and 0.37%). With increasing dietary Na or Cl levels, linear (*p* < 0.0001) and quadratic (*p* < 0.0001) increases occurred in ADG, ADFI, and average daily water consumption, while F/G exhibited a linear (*p* < 0.0001) and quadratic (*p* < 0.0001) decrease from 22 to 42 d of age in broilers. Linear (*p* < 0.02) and quadratic increases (*p* < 0.03) were found in the heart indicator, serum Cl^−^ concentrations, and blood Na^+^ and Cl^−^ concentrations, whereas the kidney indicator, blood K^+^ concentrations, and serum K^+^, UA, and glucose (GLU) concentrations showed a linear (*p* < 0.04) and quadratic (*p* < 0.004) decrease with an increase in dietary Na or Cl levels. The excreta water content, liver indicator, and jejunal crypt depth also showed a linear increase (*p* < 0.02). The ideal levels of dietary Na or Cl were estimated to be 0.07% to 0.12%, based on the most suitable broken-line and asymptotic models (*p* ≤ 0.0002) using those sensitive indicators mentioned above. It was suggested that the suitable dietary Na or Cl level of 0.12% would adequately fulfill the Na or Cl metabolic demands of chickens from 22 to 42 d of age.

## Simple Summary

The current study identified average daily gain (ADG), average daily feed intake (ADFI), feed-to-gain ratio (F/G), blood Na^+^, K^+^, Cl^−^ concentrations, and serum K^+^ and uric acid (UA) concentrations as sensitive indicators for determining the ideal level of sodium (Na) or chlorine (Cl) in a corn–soybean meal diet for chickens from 22 to 42 d of age. The dietary Na or Cl content of 0.12% in the corn–soybean meal diet was recommended to satisfy all the aforementioned metabolic needs related to Na or Cl.

## 1. Introduction

Both sodium (Na) and chlorine (Cl) are vital electrolytes in animal bodies, playing crucial roles in maintaining osmotic pressure and creating a suitable metabolic environment [1]. The ninth revised edition of *Nutrient Requirements for Poultry* [2] set up nutrient requirement values based on recommendations from reputable journals to prevent the use of commercial data. Furthermore, studies often employed semi-purified or purified diets as the content of Na or Cl in conventional feedstuff is uncertain.

In the past 30 years, due to genetic progress in poultry breeding, the ideal dietary Na or Cl level for broilers has undergone changes. Early researchers studied the effect of different Na:Cl ratios on the performances of broilers and found that broilers at a Na:Cl ratio of 1:1 could achieve the best growth performance [3]. Therefore, both the NRC [2] and the Chicken Feeding Standard of China (2004) [4] proposed dietary Na or Cl requirements of chickens under the Na:Cl ratio of 1:1. Zhang et al. [5] from our laboratory studied an ideal dietary Na or Cl requirement under the Na:Cl ratio of 1:1 for chickens from 1 to 21 d of age, and found that 0.16% would be the ideal dietary Na or Cl concentration for these chickens given a corn–soybean meal-based diet, which is lower than the recommendation at 0.20% by the NRC [2] and the Chicken Feeding Standard of China [4]. The NRC [2] and the Chicken Feeding Standard of China [4] proposed a dietary Na or Cl demand of 0.15% under a Na:Cl ratio of 1:1 for broilers between 22 and 42 d of age. Hurwitz et al. [3] observed that ideal weight gain in broilers during d 22 to 35 was achieved at 0.16% Na and 0.17% Cl under a dietary Na:Cl ratio of approximately 1:1. Jiang et al. [6] reported that yellow-feathered chickens fed with a diet of 0.10% Na and 0.11% Cl during the 22–42 d of age showed the best growth performance. Differences observed in the aforementioned studies concerning dietary Na or Cl requirements might be attributed to variations in chicken breeds, evaluation indicators, dietary formulations, environmental conditions, and so on.

Based on the above research advances, our hypothesis was that when the Na:Cl ratio was 1:1 in the diet, the ideal dietary Na or Cl concentration for chickens given a corn–soybean meal diet between 22 and 42 d of age might differ from dietary Na or Cl demand of 0.15% proposed by the NRC [2] and the Chicken Feeding Standard of China [4]. Therefore, in order to verify the aforementioned hypothesis, the aim of this study was to investigate the impact of dietary Na or Cl concentrations, while maintaining a constant Na:Cl ratio of 1:1, on growth performance, water consumption, and the excreta moisture content, organ indices, blood gas indicators, serum indicators, and the jejunal morphological characteristics of broilers so as to find the sensitive evaluating indicators and select their best fitted mathematical models to estimate the ideal dietary Na or Cl level of broilers fed the corn–soybean meal diet from 22 to 42 d of age.

## 2. Materials and Methods

### 2.1. Experimental Design and Treatments 

In a completely randomized design, under the condition of a dietary Na:Cl ratio of 1:1, 6 treatments of 6 different dietary Na or Cl levels [0.02% Na and 0.07% Cl (the control with no added Na or Cl), 0.13%, 0.19%, 0.25%, 0.31% and 0.37%] were designed around the dietary Na or Cl requirement of 0.15% proposed by the NRC [2] and the Chicken Feeding Standard of China [4].

### 2.2. Birds and Diets

One-day-old Arbor Acres (AA) male broiler chicks were purchased from Jinghai Poultry Group (Nantong, Jiangsu, China). During 1–21 d of age, all of the male broilers of AA were given the same corn–soybean meal complete diet. On day 22, 288 healthy and similar-weight AA male chickens were selected and randomly assigned to one of six treatments. Each treatment comprised eight replicate cages, with six chickens in every cage. The broilers were housed in stainless steel cages (90 cm in length × 70 cm in width × 45 cm in height) with 16 h of incandescent lighting and 8 h of darkness per day. Cleanliness, hygiene, appropriate temperature, and good ventilation were maintained in the house according to the AA Broiler Management Guidelines [7]. Birds were given free access to feed and tap water which contained quite a low concentration (less than 44 μg/mL) of calcium, Na, or potassium (K). Daily records were kept of water consumption and the number of deceased birds in each replicate cage. On d 42, after overnight fasting, broiler weights and feed intakes were accurately measured using an electronic scale for each individual cage. And finally, the average daily gain (ADG), average daily feed intake (ADFI), feed-to-gain ratio (F/G), average daily water consumption, and mortality according to the replicated cage were computed for the broilers from 22 to 42 d of age.

The complete diet during d 1–21 and the corn–soybean meal basal diet from 22 to 42 d of age were formulated to fulfill or surpass the needs of all nutrients during d 1–21 and all other nutrients except for Na or Cl during d 22–42 as proposed by the NRC [2] and the Chicken Feeding Standard of China [4] (Table 1).

All of the other 5 treatment diets except for the control basal diet were formulated by adding different amounts of sodium chloride (NaCl) and sodium bicarbonate (NaHCO_3_) from Guoyao Company (Shanghai, China) (both of reagent grade) into the basal diet, replacing an equivalent weight of feed-grade powdered zeolite. The NaCl had a purity level of 99.5%, containing 39.1% Na and 60.4% Cl, while the NaHCO_3_ also had a purity of 99.5% with a Na content of 27.2%. The determined Na contents in all treatment diets are shown in Table 2. All birds were fed the treatment diets in the form of mash.

### 2.3. Sample Collections and Preparations

Diet samples were collected to analyze the contents of dietary CP, Ca, Na, Cl, and K. From 37 to 41 d of age, all the excreta from each replicate cage were collected and weighed at 14:00 every day. After mixing, 10% of the total weight of excreta was randomly collected and stored in a −20 °C freezer for further measurement of water content.

On d 42, two birds from each replicate cage (sixteen birds/treatment) were chosen according to the cage average BW. The 1 mL blood in a heparin lithium anticoagulation tube was obtained from the wing vein of one broiler for blood gas analyses. The blood samples without anticoagulant were taken from the wing veins of the above two broilers. After leaving the blood to rest for 30 min at room temperature, it was centrifuged at a speed of 3000× *g* for 15 min to obtain the serum. The serum was transferred to a 2 mL EP tube and stored at −20 °C for analyses of Na^+^, K^+^, Cl^−^, uric acid (UA), and glucose (GLU) concentrations. Before analyses, serum samples from two broilers per replicate cage were mixed into one sample in the same proportion. 

After bleeding, one bird of the above two birds was slaughtered by cervical dislocation and dissected to isolate the heart, liver (excluding gallbladder), spleen, lung, and kidney, and then these organs were weighed to compute organ indicators [organ index (g/kg) = weight of internal organs (g)/live weight (kg)]. And then, the jejunal segment was taken from the above-killed bird, fixed in a 4% paraformaldehyde solution, and processed for routine tissue sectioning for the measurement of the jejunal morphology.

### 2.4. Measurements of CP, Ca, Na, Cl, and K Contents in Diets and Tap Water as Well as Excreta Moisture Content

Dietary CP content was analyzed according to the method of the national standard [8]. Calcium, Na, and K contents in diets and tap water were measured according to a previous method [9]. The Cl concentrations in diets and tap water were assayed using three different methods: inductively coupled plasma mass spectrometry [10], titration with AgNO_3_ [11], and an ion-selective electrode [12]. Unfortunately, the analyzed results showed significant variations for reasons that remain unclear, leading to our inability to present the Cl concentrations in diets and tap water. The water content in the excreta was measured using the national standard [13]. The formula for computing the water content in the excreta is as follows: excreta water content (%) = (weight of excreta before drying−weight of excreta after drying)/weight of excreta before drying × 100.

### 2.5. Measurements of Blood Gas and Serum Parameters

Blood samples were analyzed for partial pressure of carbon dioxide (PCO_2_), pH, total carbon dioxide (TCO_2_), base excess (BE), anion gap (AG), H^+^, HCO_3_^−^, Na^+^, Cl^−^, and K^+^ using a blood gas analyzer (VS4–6163, IDEXX VetStat, Westbrook, ME, USA). Serum concentrations of K^+^, Na^+^, Cl^−^, GLU, and UA were measured using a biochemical analyzer (7180, Hitachi, Tokyo, Japan). Serum osmotic pressure (OSM) was then calculated as OSM = 2 × Na + 2 × K + GLU + UA, where OSM mainly originated from dissolved crystal substances.

### 2.6. Measurement of the Jejunal Morphology

The jejunal samples were treated according to the description of Yang et al. [14]. Hematoxylin–eosin staining was used to stain the sections, which were then sealed. The sealed sections were observed under a computer-aided optical microscope (CKX53, OLYMPUS, Tokyo, Japan) at ×40 magnification. ImageJ 2 software was employed to assess the height of villi (VH) and the depth of crypts (CD). The VH refers to the vertical height from the top of the villi to the opening of the crypts, while CD refers to the distance from the base opening between two villi to the bottom of the intestinal gland. The VH/CD ratio was then calculated [15].

### 2.7. Statistical Analyses

The data obtained from this experiment were analyzed by a one-way analysis of variance using the general linear model in SAS 9.4 [16]. The least significant difference method was used to test discrepancies among the means. Mortality data were statistically analyzed after arcsine transformation. The linear or quadratic responses of responsive indices to dietary levels of Na or Cl were examined by the orthogonal comparison method [17,18]. Regression analyses of broken-line, asymptotic, and quadratic models were performed, and the most suitable models were used to determine the ideal dietary levels of Na or Cl (the break point from the broken-line model or 95% of the maximum response from the asymptotic model) for broiler chicks [19,20]. The best fitted models were selected based on the following three criteria, the smallest *p* value, the largest coefficient of determination (R^2^), and the smallest mean square error of the residuals, because a smaller *p* value, a larger R^2^, and a smaller mean square error of the residuals all indicate a higher credibility of the model. Each replicate cage served as an experimental unit, and the statistical significance was set at *p* < 0.05.

## 3. Results

### 3.1. Growth Performance, Mortality, Average Daily Water Consumption, and Excreta Moisture Content

Dietary Na or Cl level did not influence (*p* > 0.70) the mortality of broilers from 22 to 42 d of age but had an effect (*p* < 0.0001) on ADG, ADFI, F/G, average daily water consumption (d 22 to 41), and excreta moisture content (d 37 to 42) of broilers (Table 3). 

As dietary Na or Cl levels rose, there was a linear (*p* < 0.0001) and quadratic (*p* < 0.0001) increase in ADG, ADFI, and average daily water consumption, while the F/G ratio of broilers showed a linear (*p* < 0.0001) and quadratic (*p* < 0.0001) decrease, and excreta moisture content increased linearly (*p* < 0.0001).

### 3.2. Organ Indices

The level of dietary Na or Cl did not influence (*p* > 0.16) the spleen and lung indicators in 42-day-old broilers but had an effect (*p* < 0.03) on the heart, liver, and kidney indicators (Table 4). As dietary Na or Cl levels rose, there was a linear (*p* < 0.002) and quadratic (*p* < 0.006) increase in the heart indicator, while the kidney indicator showed a linear (*p* < 0.04) and quadratic (*p* < 0.004) decrease, and the liver indicator increased linearly (*p* < 0.001).

### 3.3. Blood Gas Indicators

The level of dietary Na or Cl did not influence (*p* > 0.14) the blood pH, PCO_2_, HCO_3_^−^, TCO_2_, BE, H^+^, and AG in 42-day-old broilers but had an effect (*p* < 0.0001) on blood Na^+^, Cl^−^, and K^+^ concentrations (Table 5). As dietary Na or Cl levels rose, there was a linear (*p* < 0.0001) and quadratic (*p* < 0.002) increase in blood Na^+^ and Cl^−^ concentrations, while blood K^+^ concentration showed a linear (*p* < 0.0001) and quadratic (*p* < 0.0001) decrease. 

### 3.4. Serum Physiological and Biochemical Indicators

The level of dietary Na or Cl did not influence (*p* > 0.70) the serum Na^+^ concentration and OSM in 42-day-old broilers but had an effect (*p* < 0.03) on serum K^+^, Cl^−^, GLU, and UA concentrations (Table 6). As dietary Na or Cl levels rose, there was a linear (*p* < 0.02) and quadratic (*p* < 0.03) increase in serum Cl^−^ concentration, while serum K^+^, GLU, and UA concentrations showed a linear (*p* < 0.005) and quadratic (*p* < 0.004) decrease. 

### 3.5. Jejunal Morphology

The level of dietary Na or Cl did not influence (*p* > 0.11) the VH and VH/CD ratio of the jejunum in 42-day-old broilers but had an effect (*p* < 0.03) on the CD of the jejunum (Table 7). As dietary Na or Cl levels were up, jejunal mucosal CD increased linearly (*p* < 0.02).

### 3.6. Estimation of Ideal Dietary Na or Cl Levels

Utilizing the data from the above findings and the most suitable broken-line or asymptotic models (*p* ≤ 0.0002, R^2^ = 0.4897−0.9753, Table 8), eight crucial parameters were identified for assessing the ideal levels of Na or Cl in the corn–soybean meal diet for broilers from 22 to 42 d of age. These parameters include ADG, ADFI, and F/G, as well as blood Na^+^, K^+^, Cl^−^ concentrations, and serum K^+^ and UA concentrations. According to the most appropriate broken-line or asymptotic models for these sensitive parameters, the estimated ideal dietary concentrations of Na or Cl for these chickens ranged from 0.07% to 0.12%, as detailed in Table 8.

## 4. Discussion

The present study has revealed that ADG, ADFI, and F/G, as well as blood Na^+^, K^+^, Cl^−^ concentrations, serum K^+^ levels, and UA concentrations, served as sensitive indicators for determining the ideal Na or Cl level in the corn–soybean meal diet for broilers aged 22 to 42 d. Based on the most suitable models fitted for these indicators, the estimated ideal dietary Na or Cl level for these broilers ranged from 0.07% to 0.12%. Consequently, the dietary Na or Cl concentration of 0.12% in the corn–soybean meal diet was recommended to fulfill all of the above-related metabolic needs for Na or Cl, which is lower than the suggested level of 0.15% by the NRC [2] and the Chicken Feeding Standard of China [4]. These findings have supported our hypothesis and provided a scientific experimental basis for the rational supplementation of Na or Cl in chicken production.

In previous reports, dietary Na or Cl levels affected the growth performance of broilers. Hurwitz et al. [3] reported that when the concentrations of Na or Cl in the diet were 0.16% and 0.17%, respectively, under a Na:Cl ratio of approximately 1:1, these Na or Cl levels supported the maximal weight gain of broilers during d 21 to 35. In the current study, there was a linear and quadratic increase in the BW, ADG, and ADFI of chickens aged 22 to 42 d as the dietary concentrations of Na or Cl were elevated. Studies indicated that increasing dietary Na levels increased the water intake and the excreta water content of broilers [21,22,23]. Oviedo-Rondon et al. [24] found that reducing the dietary Na intake of broilers was essential for effectively managing litter wetness. The same findings were observed in our current research that the average daily water consumption and excreta water content of broilers increased with an increase in dietary Na or Cl concentration. Nevertheless, utilizing these two indicators for assessing the ideal dietary requirement of chickens for Na or Cl is inadequate, as they do not facilitate water conservation or effective manure/litter moisture management.

Previous studies indicated that raising the level of Na or Cl in the diet led to a reduction in the indices of the liver, heart, and kidney in chickens [25]. In the same manner, in our present research, as the dietary levels of Na or Cl rose, a linear and quadratic decline was observed in the heart and kidney indices of chickens. As the coefficients of determination (R^2^) of the best fitted asymptotic models of heart indicator (0.3125) and kidney indicator (0.2591) were too low, these two indicators were unsuitable for estimating the ideal dietary Na or Cl concentration of chickens from 22 to 42 d of age.

In this present experiment, as dietary Na or Cl levels rose, the concentrations of blood Na^+^ or Cl^−^ increased linearly and quadratically in 42-day-old broilers. However, other blood gas parameters did not change significantly possibly because the body regulated blood pH through ion buffering and renal excretory function [26]. The results mentioned above align with those presented by Murakami et al. [27]. Accordingly, increasing the intake of Na or Cl could lead to higher blood Na^+^ and Cl^−^ concentrations in broilers.

Both Na and Cl are important components of the blood crystal osmotic pressure, which usually maintains the internal environment in a relatively stable state. Increasing salt intake increased serum concentrations of Na^+^ and UA, which could further cause changes in serum ion concentrations, GLU, and OSM in humans [28]. Jiang et al. [6] reported that increasing dietary Na or Cl levels significantly increased serum Na^+^ and Cl^−^ concentrations, and OSM, but significantly reduced serum K^+^ and GLU concentrations in yellow-feathered chickens. In the present experiment, the concentrations of serum K^+^, GLU, and UA showed a linear and quadratic decrease, while Cl^−^ concentration showed a linear and quadratic increase in broilers. However, increasing dietary Na or Cl levels did not significantly affect serum OSM and Na^+^ concentrations. Some disparities from the above results might be due to differences in animal breed, diet composition, feeding environment, and so on. As the coefficients of determination (R^2^) of the best fitted asymptotic models of serum Cl^−^ (0.2591) and GLU (0.2591) were too low, these two indicators were unsuitable for estimating the ideal dietary Na or Cl concentration of chickens from 22 to 42 d of age.

The VH and CD of the small intestine are important indicators for evaluating the small intestine’s digestive function [29]. The VH/CD ratio can reflect the ability of intestinal digestion and absorption, among which the villus epithelial cells play a role mainly in absorption, while the crypt epithelial cells play a role mainly in secretion [30]. In the present study, we found that dietary Na or Cl levels did not affect VH and the VH/CD ratio in the jejunum of 42-day-old chickens, but with increasing dietary Na or Cl levels, CD showed a linear increase, and thus this indicator was unsuitable for judging the ideal dietary Na or Cl concentration of chickens from 22 to 42 d of age. These findings align with the results of Chen et al. [31], who also demonstrated a notable effect of dietary Na or Cl levels on the CD of the jejunum in broilers.

In addition, it should be pointed out that the results of both linear and quadratic analyses for most indices are highly affected by the results of the negative control, majorly due to the limited number of treatments set below the standard of 0.15%. Therefore, additional treatments with lower levels of Na or Cl for broilers should be designed in future research studies.

## 5. Conclusions

Based on the best fitted broken-line or asymptotic models utilizing sensitive indicators such as ADG, ADFI, F/G, blood Na^+^, K^+^, and Cl^−^ concentrations, and serum K^+^ and UA concentrations, the ideal dietary levels of Na or Cl were estimated to range between 0.07% and 0.12%. It was recommended that the dietary Na or Cl level of 0.12% would sufficiently meet the metabolic requirements of chickens from 22 to 42 d of age.

## Figures and Tables

**Table 1:** Composition and nutrient levels of the complete diet (d 1–21) and the basal diet (d 22–42) for broilers (as-fed basis).

Items	D 1 to 21	D 22 to 42
Ingredients, %		
Corn, 8% CP	53.70	57.24
Soybean meal, 46% CP	37.45	32.85
Soybean oil	4.75	5.55
CaHPO_4_ ^1^	1.95	1.70
Limestone ^1^	1.24	1.09
Salt ^1^	0.30	0.00
DL-Met ^1^	0.32	0.16
Vitamin–mineral premix ^2,3^	0.29	0.21
Zeolite powder ^1,4^	0.00	1.20
Total	100.00	100.00
Nutrient levels, %		
Metabolizable energy, MJ/kg	12.64	12.94
CP ^5^	21.39	19.98
Lys	1.13	1.01
Met	0.61	0.44
Thr	0.80	0.73
Trp	0.23	0.21
Met + cys	0.92	0.72
Ca ^5^	1.04	0.89
Nonphytate P	0.45	0.40
Na ^5^	0.12	0.02
Cl	0.22	0.07
K ^5^	1.06	0.97

^1^ Feed grade. ^2^ Provided per kilogram of diet for d 1 to 21: VA 12,000 IU, VD_3_ 4500 IU, VE 33 IU, VK_3_ 3 mg, VB_1_ 3 mg, VB_2_ 9.6 mg, VB_6_ 4.5 mg, VB_12_ 0.03 mg, pantothenic acid calcium 15 mg, niacin 54 mg, folic acid 1.5 mg, biotin 0.15 mg, choline 700 mg, Cu (CuSO_4_·5H_2_O) 6 mg, Fe (FeSO_4_·H_2_O) 40 mg, Zn (ZnSO_4_·H_2_O) 60 mg, Mn (MnSO_4_·H_2_O) 110 mg, Se (Na_2_SeO_3_) 0.35 mg, I (Ca(IO_3_)_2_·H_2_O) 0.35 mg. ^3^ Provided per kilogram of diet for d 22 to 42: VA 8000 IU, VD_3_ 3000 IU, VE 22 IU, VK_3_ 2 mg, VB_1_ 2 mg, VB_2_ 6.4 mg, VB_6_ 3 mg, VB_12_ 0.02 mg, pantothenic acid calcium 10 mg, niacin 36 mg, folic acid 1.0 mg, biotin 0.10 mg, choline 500 mg, Cu (CuSO_4_·5H_2_O) 6 mg, Fe (FeSO_4_·H_2_O) 30 mg, Zn (ZnSO_4_·H_2_O) 40 mg, Mn (MnSO_4_·H_2_O) 80 mg, Se (Na_2_SeO_3_) 0.35 mg, I (Ca(IO_3_)_2_·H_2_O) 0.35 mg. ^4^ NaCl or NaHCO_3_ supplements were added in place of equivalent weights of powdered zeolite. ^5^ Determined values.

**Table 2:** Analyzed Na concentrations in diets for chickens between 22 and 42 d of age.

Dietary Na or Cl Levels, %	Analyzed Dietary Na Contents ^1^, %
0.02 Na and 0.07 Cl, control	0.02
0.13	0.12
0.19	0.17
0.25	0.24
0.31	0.30
0.37	0.36

^1^ Values of analyzed Na contents based on triplicate determinations.

**Table 3:** Impact of dietary Na or Cl concentration on growth performance, mortality, average daily water consumption, and excreta moisture content of chickens from 22 to 42 d of age ^1^.

Dietary Na or Cl, %	ADG, g/d	ADFI, g/d	F/G, g/g	Mortality ^2^, %	Average Daily Water Consumption, mL/d	Excreta Moisture Content, %
0.02 Na and 0.07 Cl, control	29.4 ^c^	92.5 ^b^	3.06 ^a^	2.09	221 ^d^	80.0 ^c^
0.13	84.5 ^ab^	144 ^a^	1.70 ^b^	2.09	343 ^c^	82.2 ^b^
0.19	84.5 ^ab^	141 ^a^	1.69 ^b^	0.00	355 ^bc^	83.1 ^b^
0.25	87.3 ^a^	144 ^a^	1.65 ^b^	2.09	360 ^bc^	82.6 ^b^
0.31	85.4 ^a^	145 ^a^	1.70 ^b^	0.00	374 ^ab^	84.1 ^ab^
0.37	84.0 ^b^	142 ^a^	1.69 ^b^	0.00	395 ^a^	85.1 ^a^
Pooled SE	1.21	2.2	0.054	1.482	10.3	0.72
*p* value						
Dietary Na or Cl	<0.0001	<0.0001	<0.0001	0.7001	<0.0001	<0.0001
Linear	<0.0001	<0.0001	<0.0001		<0.0001	<0.0001
Quadratic	<0.0001	<0.0001	<0.0001		<0.0001	0.8358

ADG = average daily gain; ADFI = average daily feed intake; F/G = feed/gain ratio. ^a–d^ Values with different superscripts within the same column differ (*p* < 0.05). ^1^ Values are expressed as the means of 8 replicate cages of 6 birds per replicate cage (*n* = 8). ^2^ Percentage data for the mortality of birds were transformed to arcsine for analysis.

**Table 4:** Impact of dietary Na or Cl concentration on organ indices of chickens at 42 d of age ^1^.

Dietary Na or Cl, %	Heart Indicator, g/kg	Liver Indicator, g/kg	Spleen Indicator, g/kg	Lung Indicator, g/kg	Kidney Indicator, g/kg
0.02 Na and 0.07 Cl, control	4.49 ^c^	16.5 ^c^	1.18	6.96	4.14 ^a^
0.13	5.31 ^ab^	17.9 ^bc^	1.18	6.01	3.48 ^b^
0.19	5.50 ^a^	19.0 ^ab^	1.46	6.51	3.12 ^b^
0.25	5.05 ^b^	18.6 ^ab^	1.36	5.73	3.32 ^b^
0.31	5.33 ^ab^	18.7 ^ab^	1.35	6.88	3.26 ^b^
0.37	5.26 ^ab^	19.6 ^a^	1.39	5.72	3.67 ^ab^
Pooled SE	0.151	0.61	0.110	0.432	0.204
*p* value					
Dietary Na or Cl	0.0002	0.0103	0.3139	0.1632	0.0221
Linear	0.0011	0.0005			0.0324
Quadratic	0.0052	0.3005			0.0032

^a–c^ Values with different superscripts within the same column differ (*p* < 0.05). ^1^ Values are expressed as the means of 8 replicate cages of 1 bird per replicate cage (*n* = 8).

**Table 5:** Impact of dietary Na or Cl concentration on blood gas indicators of chickens at 42 d of age ^1^.

Dietary Na or Cl, %	pH	PCO_2_, mmHg	HCO_3_^−^, mmol/L	TCO_2_, mmol/L	BE, mmol/L	Na^+^, mmol/L	Cl^−^, mmol/L	H^+^, nmol/L	AG, mmol/L	K^+^, mmol/L
0.02 Na and 0.07 Cl, control	7.38	39.0	20.7	21.7	−1.76	131 ^b^	107 ^b^	42.0	13.2	8.26 ^a^
0.13	7.40	37.0	21.2	22.2	−1.00	147 ^a^	116 ^a^	40.4	15.6	4.96 ^b^
0.19	7.36	41.6	21.3	22.4	−1.30	148 ^a^	115 ^a^	43.5	16.1	5.00 ^b^
0.25	7.39	38.3	20.7	21.7	−1.56	144 ^a^	115 ^a^	41.1	13.2	5.10 ^b^
0.31	7.40	38.1	20.9	21.9	−1.23	147 ^a^	116 ^a^	40.5	14.9	5.13 ^b^
0.37	7.41	35.6	19.9	20.9	−1.53	145 ^a^	117 ^a^	40.7	13.8	5.11 ^b^
Pooled SE	0.011	1.52	0.59	0.64	0.520	2.1	1.1	1.21	1.42	0.230
*p* value										
Dietary Na or Cl	0.3867	0.1421	0.6283	0.6063	0.9258	<0.0001	<0.0001	0.4664	0.5139	<0.0001
Linear						<0.0001	<0.0001			<0.0001
Quadratic						<0.0001	0.0018			<0.0001

PCO_2_ = partial pressure of CO_2_; TCO_2_ = total CO_2_; BE = base excess; AG = anion gap. ^a,b^ Values with different superscripts within the same column differ (*p* < 0.05). ^1^ Values are expressed as the means of 8 replicate cages of 1 bird per replicate cage (*n* = 8).

**Table 6:** Impact of dietary Na or Cl concentration on serum physiological and biochemical parameters of chickens at 42 d of age ^1^.

Dietary Na or Cl, %	Na^+^, mmol/L	K^+^, mmol/L	Cl^−^, mmol/L	GLU, mmol/L	UA, mmol/L	OSM, mmol/L
0.02 Na and 0.07 Cl, control	164	2.40 ^a^	43.7 ^b^	10.5 ^a^	0.512 ^a^	344
0.13	183	1.50 ^b^	46.3 ^a^	6.95 ^b^	0.197 ^b^	375
0.19	184	1.65 ^b^	46.5 ^a^	6.56 ^b^	0.203 ^b^	378
0.25	174	1.42 ^b^	45.9 ^a^	7.72 ^b^	0.200 ^b^	359
0.31	173	1.55 ^b^	46.0 ^a^	7.57 ^b^	0.217 ^b^	356
0.37	173	1.65 ^b^	46.2 ^a^	7.02 ^b^	0.253 ^b^	356
Pooled SE	10.1	0.122	0.60	0.671	0.0361	19.0
*p* value						
Dietary Na or Cl	0.7016	<0.0001	0.0254	0.0019	<0.0001	0.8181
Linear		<0.0001	0.0110	0.0045	<0.0001	
Quadratic		<0.0001	0.0272	0.0032	<0.0001	

GLU = glucose; UA = uric acid; OSM = osmotic pressure. ^a,b^ Values with different superscripts within the same column differ (*p* < 0.05). ^1^ Values are expressed as the means of 8 replicate cages of 2 birds per replicate cage (*n* = 8).

**Table 7:** Impact of dietary Na or Cl concentration on the jejunal mucosal morphology of chickens at 42 d of age ^1^.

Dietary Na or Cl, %	Jejunal VH, μm	Jejunal CD, µm	VH/CD, µm/µm
0.02 Na and 0.07 Cl, control	1059	91 ^b^	11.9
0.13	1067	103 ^a^	10.8
0.19	1136	104 ^a^	11.4
0.25	954	107 ^a^	9.2
0.31	1082	112 ^a^	10.3
0.37	934	101 ^ab^	10.8
Pooled SE	60.0	4.0	0.70
*p* value			
Dietary Na or Cl	0.1770	0.0240	0.1158
Linear		0.0121	
Quadratic		0.0695	

VH = villus height; CD = crypt depth. ^a,b^ Values with different superscripts within the same column differ (*p* < 0.05). ^1^ Values are expressed as the means of 8 replicate cages of 1 bird per replicate cage (*n* = 8).

**Table 8:** The ideal dietary Na or Cl level of chickens from 22 to 42 d of age as estimated based on the best fitted models.

Dependent Variable	Regression Equation ^1^	Coefficient of Determination (R^2^)	*p* Value	Ideal Dietary Na or Cl Levels, %
ADG	Y = 19 + 562X (0.02 ≤ X ≤ 0.12)	0.9753	<0.0001	0.12
	Y = 20 + 568X (0.12 ≤ X ≤ 0.36)			
ADFI	Y = 82 + 510X (0.02 ≤ X ≤ 0.12)	0.9409	<0.0001	0.12
	Y = 82 + 511X (0.12 ≤ X ≤ 0.36)			
F/G	Y = 3.31 − 13.92X (0.02 ≤ X ≤ 0.12)	0.9461	<0.0001	0.12
	Y = 3.30 − 13.99X (0.12 ≤ X ≤ 0.36)			
Blood Na^+^	Y = 124 + 55X (0.02 ≤ X ≤ 0.09)	0.5810	<0.0001	0.09
	Y = 149 + 114X (0.09 ≤ X ≤ 0.36)			
Blood Cl^−^	Y = 123 + 19X (0.02 ≤ X ≤ 0.07)	0.5855	<0.0001	0.07
	Y = 122 + 20X (0.07 ≤ X ≤ 0.36)			
Blood K^+^	Y = 1.79 + 16.29 × e^−64.33X^	0.7991	<0.0001	0.08
Serum K^+^	Y = 2.57 − 9.26X (0.02 ≤ X ≤ 0.11)	0.4897	<0.0001	0.11
	Y = 2.54 − 9.54X (0.11 ≤ X ≤ 0.36)			
Serum UA	Y = 0.57 − 3.21X (0.02 ≤ X ≤ 0.12)	0.5834	0.0002	0.12
	Y = 0.54 − 3.46X (0.12 ≤ X ≤ 0.36)			

ADG = average daily gain; ADFI = average daily feed intake; F/G = feed/gain ratio; UA = uric acid; ^1^ Y = measurement of index; X = dietary analyzed Na concentration (%).

## Data Availability

The data presented in this study are available in the article.
